# Genetic Alterations of the Thrombopoietin/MPL/JAK2 Axis Impacting Megakaryopoiesis

**DOI:** 10.3389/fendo.2017.00234

**Published:** 2017-09-12

**Authors:** Isabelle Plo, Christine Bellanné-Chantelot, Matthieu Mosca, Stefania Mazzi, Caroline Marty, William Vainchenker

**Affiliations:** ^1^INSERM UMR 1170, Gustave Roussy, Villejuif, France; ^2^Université Paris-Saclay, UMR1170, Gustave Roussy, Villejuif, France; ^3^Gustave Roussy, UMR1170, Villejuif, France; ^4^Department of Genetics, AP-HP Hôpitaux Universitaires Pitié Salpêtrière - Charles Foix, UPMC Univ Paris 06, Paris, France; ^5^Université Paris-Diderot, Paris, France

**Keywords:** thrombopoietin, MPL, JAK2, MPLR102P, thrombocytosis, thrombocytopenia

## Abstract

Megakaryopoiesis is an original and complex cell process which leads to the formation of platelets. The homeostatic production of platelets is mainly regulated and controlled by thrombopoietin (TPO) and the TPO receptor (MPL)/JAK2 axis. Therefore, any hereditary or acquired abnormality affecting this signaling axis can result in thrombocytosis or thrombocytopenia. Thrombocytosis can be due to genetic alterations that affect either the intrinsic MPL signaling through gain-of-function (GOF) activity (*MPL, JAK2, CALR*) and loss-of-function (LOF) activity of negative regulators (*CBL, LNK*) or the extrinsic MPL signaling by *THPO* GOF mutations leading to increased TPO synthesis. Alternatively, thrombocytosis may paradoxically result from mutations of *MPL* leading to an abnormal MPL trafficking, inducing increased TPO levels by alteration of its clearance. In contrast, thrombocytopenia can also result from LOF *THPO* or *MPL* mutations, which cause a complete defect in MPL trafficking to the cell membrane, impaired MPL signaling or stability, defects in the TPO/MPL interaction, or an absence of TPO production.

## Introduction

Megakaryopoiesis is an original cell process which leads to the formation of platelets. The homeostatic production of platelets is mainly regulated by thrombopoietin (TPO) and the TPO receptor (MPL)/JAK2 axis. TPO regulates nearly all stages of the megakaryocyte (MK) differentiation, and this explains that numerous diseases characterized by an alteration in MK/platelet production leading to thrombocytopenia or thrombocytosis are due to either acquired or hereditary mutations in *MPL, THPO*, or *JAK2*.

## Importance of the TPO/MPL/JAK2 Axis in Megakaryopoiesis

### Megakaryopoiesis

Megakaryopoiesis is the process leading to the differentiation of bone marrow progenitors to MKs, giving rise to circulating platelets in the blood. The first step requires the commitment of a multipotent hematopoietic stem cell (HSC) to an MK progenitor followed by several proliferation and differentiation steps (1). There is increasing evidence that MK progenitors may arise directly from an HSC or from a MK/erythroid progenitor (2). This may explain that numerous transcription factors such as TAL1, GATA2, ERG/FLI-1, and RUNX1 regulate HSC properties and MK differentiation and that others, such as GATA1 and GFI1b, regulate also erythroid and MK differentiations. Moreover, TPO plays a central role not only in the MK/platelet lineage but also in HSCs (3). MK progenitors first proliferate before switching to endomitosis, which is a mitosis without cytokinesis (4). Polyploidization generates MKs with a modal distribution with a major peak at 16N. MKs further mature by increasing their cytoplasm and membrane to finally fragment to give rise to platelets. The fragmentation is a dynamic and regulated process. In the human adult, most of megakaryopoiesis takes place in the bone marrow, except the platelet release. Either MKs will send long pseudopods (proplatelets) through the endothelial barrier that will fragment into platelets in the blood flow (5) or the entire MKs will migrate into the circulation to fragment into platelets inside the lung microcirculation (6). It has also been underscored that mouse megakaryopoiesis may occur in the parenchymal lung (6).

### TPO/MPL/JAK2 Axis

**Figure 1:**
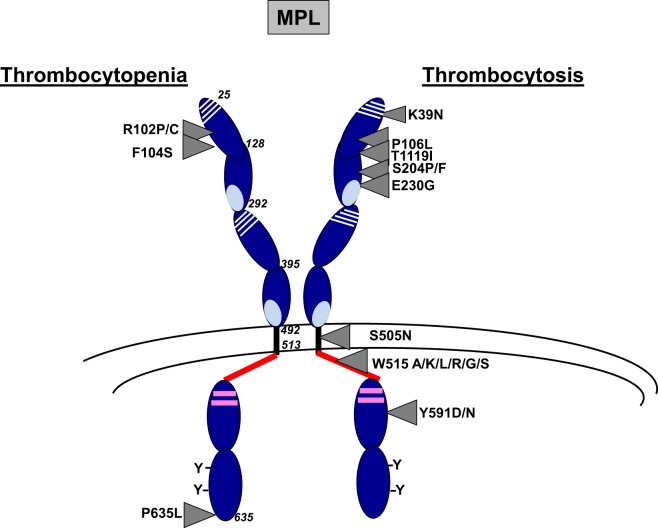
Main MPL mutations resulting in thrombocytosis or thrombocytopenia. MPL is composed of three main domains including extracellular (25–491), transmembrane, (492–513) and intracytoplasmic (514–635) domains. The extracellular domain is composed of two cytokine receptor domains (CRM) that correspond to an immunoglobulin fold of fibronectin type III. The first part of the CRM has four characteristically spaced cysteine residues, and the second part displays a WSXWS motif at the C-terminal. The intracellular domain contains box1 and box2 (in pink) and several conserved tyrosine (Y) residues. Main *MPL* mutations in thrombocytosis and thrombocytopenia are indicated by arrows.

*MPL* is located on chromosome 1p34 and consists of 12 exons which encodes a protein of 70 kDa predicted weight (3). In human, MPL expression increases during megakaryopoiesis from HSCs and progenitors to MKs and platelets (7). MPL is a homodimeric class I receptor with three main domains: extracellular, transmembrane, and intracellular (Figure 1). The extracellular domain is composed of two consecutive cytokine receptor motifs composed of a fibronectin-III-like domain characterized by four conserved cysteine residues and a WSXWS motif. The transmembrane domain folds as an α-helix that is necessary for the insertion of the receptor in the membrane and is followed by a juxtamembrane or amphipathic domain composed of the RWQFP sequence (8). The intracellular domain is required to mediate the signaling through its binding *via* box1 to Janus tyrosine kinase JAK2 (9, 10). JAK2 was shown to be essential for MPL phosphorylation and for activation of downstream signaling pathways. TYK2, another member of the JAK family, can bind to MPL but can be activated by TPO only in the presence of JAK2. Thus, TYK2 was shown to be neither necessary nor sufficient for MPL phosphorylation and signaling (9).

**Figure 2:**
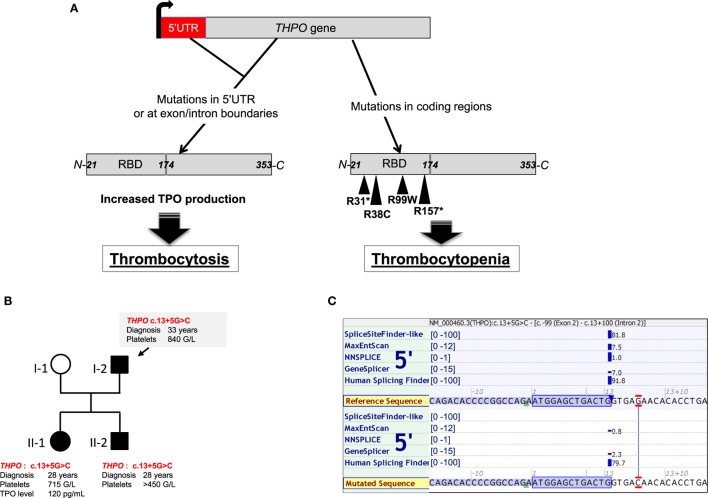
*THPO* mutations found in hereditary thrombocytosis, thrombocytopenia, and bone marrow aplasia. **(A)** Schemes of *THPO* gene and thrombopoietin (TPO) protein. Mutations in *THPO* gene affecting either 5′UTR or splice sites (defective alternative splicing) lead to increased *THPO* mRNA translation and increased TPO synthesis in hereditary thrombocytosis. Mutations in coding regions of *THPO* affect the RBD (receptor-binding domain) and are found in hereditary thrombocytopenia or bone marrow aplasia. **(B)** Illustration of a pedigree with *THPO* mutation from a family harboring a mild thrombocytosis. **(C)** *In silico* analysis of the *THPO* c.13+5G>C based on five predictive algorithms of pathogenicity for splice site defects. Analysis was run using the Alamut Visual version 2.9 software (Interactive Biosoftware, Rouen, France).

The ligand of MPL is the glycoprotein hormone TPO encoded by the *THPO* gene at chromosome 3q27.1 and composed of five coding exons. It is organized in two domains: an N-terminal cytokine domain including the first 153 amino acids followed by a highly glycosylated C-terminal domain that regulates its production and increases its half-life *in vivo* (11) (Figure 2A). TPO is constitutively produced by the liver (12). However, its level is regulated by clearance through the platelet mass (13, 14) and its synthesis by inflammation. Kidney, spleen, and bone marrow have been found to contribute to the production of TPO at a lesser extent (12). It has been suggested that the TPO produced by bone marrow stromal cells and eventually MKs play a central role in the regulation of HSC quiescence. TPO synthesis in the liver can be regulated during inflammatory conditions by stimulation of IL-6 (15). Moreover, old desialylated platelets can bind to the Ashwell–Morell receptor on hepatocytes to induce TPO gene expression through activation of the JAK/STAT3 pathway *via* IL-6 receptor activation (16).

MPL is pre-associated with JAK2 and TYK2 in the endoplasmic reticulum. The two kinases play a role of chaperone molecules and increase MPL trafficking to the cell membrane (17). The MPL/JAK2 complex becomes mature through traffic in the Golgi apparatus, where MPL undergoes successive glycosylations on asparagine residues (N-glycosylations) and is addressed at cell surface as a partially pre-dimerized receptor. TPO binding to MPL involves not only two main residues, D261 and L265, on the extracellular domain of MPL but also a site close to residue F104 (18, 19) and induces an increased dimerization of MPL and its stabilization. TPO binding induces conformational changes of MPL, which move closer the intracellular domains of the homodimer and position the two associated JAK2/TYK2 in proximity leading to their transphosphorylation (20). Active JAK2 phosphorylates several conserved tyrosine residues in the intracellular domain of MPL, which serve as docking sites for a number of substrates including STAT1, STAT3, and STAT5 and adaptors such as CBL, VAV, and SHC leading to activation of mitogen-activated protein kinase (MAPK) and phosphatidylinositol 3-kinase/AKT (21). After MPL activation, the TPO/MPL/JAK2 is internalized to either be recycled or, more often, degraded through proteasomes and lysosomes induced after activation of negative regulators such as the ubiquitin ligase CBL (22). In addition, members of the SOCS family (SOCS1, SOCS2) bind to phosphorylated JAK2 and/or MPL and induce their degradation through the proteasome pathway (23). Furthermore, LNK, an adaptor protein that binds to JAK2, and other phosphatases can limit MPL activation (24). Finally, PIAS negatively regulates the JAK2/STAT pathway by directly inhibiting the binding of STAT to DNA or by causing sumoylation of STATs (25). This negative regulation plays a central role in limiting the stimulation of MPL.

### TPO/MPL/JAK2 Axis As a Central Player in Megakaryopoiesis in Mice

The importance of the TPO/MPL/JAK2 axis in megakaryopoiesis is illustrated by several lines of evidence. Both the constitutive *thpo*^−/−^ and *mpl*^−/−^ knockout (KO) mice and the conditional *jak2* KO mice induced in adulthood after crossing them with transgenic mice expressing tamoxifen-inducible Cre under SCL promoter showed marked alterations in the HSC compartment associated with a thrombocytopenia (150–400 × 10^9^/L) (26–28). Of note, *tyk2*^−/−^ KO mice did not harbor thrombocytopenia consistently with its dispensable role in MPL activation (29). The levels of TPO were highly elevated in mouse plasma from *mpl*^−/−^ and conditional *jak2*^−/−^ mice due to the loss of clearance by platelet mass through Mpl binding. The conditional *mpl* and *jak2* KO mice were also crossed with PF4-Cre transgenic mice, which allowed the deletion of *mpl* or *jak2* in MKs and platelets, thereby only in the late stages of megakaryopoiesis. In a counterintuitive manner, these mice presented a strong thrombocytosis (5,000–10,000 × 10^9^/L) with normal TPO levels in the plasma (30, 31). Thrombocytosis in these models was explained by the stimulation of the MK progenitor proliferation still expressing Mpl. Therefore, these data showed that the proliferation of MK progenitors (first steps of megakaryopoiesis) is highly dependent on TPO in contrast to the platelet production from MKs. A strong thrombocytosis but with a partial defect in the HSC compartment was also observed in *mpl* transgenic mice performed in *mpl*^−/−^ context which leads to subnormal Mpl expression in maturing MKs and platelets. These models demonstrate that low levels of MPL in MK progenitors are sufficient to induce their proliferation and highlighted the importance of MPL expression level during megakaryopoiesis (32, 33).

## Effect of TPO/MPL/JAK2 Axis Alterations in the Stimulation of Megakaryopoiesis

*THPO*/*MPL*/*JAK2* gain-of-function (GOF) or loss-of-function (LOF) mutations as well as LOF mutations of negative regulators have been identified in diseases leading to MK hyperplasia (Table 1).

**Table 1:** Genetic alterations of the thrombopoietin (TPO)/MPL/JAK2 axis leading to thrombocytosis and thrombocytopenia.

Gene	Mutation	Somatic/germline	Mechanism		Clinical phenotype	Reference
*JAK2*	V617F	Somatic	GOF	Constitutive signaling	Thrombocytosis	(34–37)
	V617I Htz	Germline	GOF	Low constitutive signaling	Thrombocytosis	(38)
	H608N Htz		GOF			(39)
	R564Q Htz		GOF			(40)
	[S755R.R938Q] Htz		GOF			(41)
	R867Q Htz		GOF			(41)

*MPL*	W515 A/K/L/**R**/G/S	Somatic	GOF	Constitutive signaling	Thrombocytosis	(42–46)
	**S505N^a^**		GOF			(47)
	**S505N^a^** Htz	Germline	GOF	Constitutive signaling	Thrombocytosis	(44)
	K39N Htz or Hmz		LOF	Incomplete processing and reduction in MPL protein		(48)
	P106L Hmz		LOF	Reduction in MPL protein and defect of TPO clearance by platelets		(49, 50)

	R102P Hmz or C-Htz	Germline	LOF	Defective trafficking of MPL	Thrombocytopenia	(51)
	F104S Hmz or C-Htz		LOF	Normal MPL trafficking, no binding to TPO		(52)
	P635L Hmz or C-Htz		LOF	Impaired MPL stability and signaling		(51)

*THPO*	c.-31G>T Htz	Germline	GOF	Increased synthesis of TPO	Thrombocytosis	(55)
	Splice site defects Htz: (c.13+1G>C, c.141+1G>C, c.141+5G>C)		GOF			(53, 54)

	R38C^b^ Htz	Germline	LOF	Impaired TPO binding to MPL	Thrombocytopenia	(11)
	R31*^c^ Htz		LOF	No synthesis of TPO		(56)
	R99W^d^ Hmz		LOF	Impaired TPO binding to MPL		(57)
	R157*^d^ Hmz		LOF	No synthesis of TPO		(57)

*GOF, gain-of-function; LOF, loss-of-function; Htz, heterozygous state; Hmz, homozygous state; C-Htz, compound heterozygous state*.

*^a^Mutation indicated in bold involved as somatic or germline event or implicated in both phenotypes*.

*^b^Associated with bone marrow failure at homozygous state*.

*^c^Associated with inherited thrombocytopenia*.

*^d^Severe thrombocytopenia evolving to bone marrow failure*.

### Mutations on the TPO/MPL/JAK2 Axis

#### *MPL* Mutations

*MPL* mutations have been found in myeloproliferative neoplasms (MPNs) including essential thrombocythemia (ET) (3%) and primary myelofibrosis (PMF) (5%) and in hereditary thrombocytosis (Figure 1). The main mutations in MPNs are located in the exon 10 of *MPL* and affect the tryptophan at position 515 leading to amino acid changes such as *MPL* W515K/L/A/G/S/R that show a GOF activity (42–46). The tryptophan at position 515 is part of the amphipathic domain located at the junction between the transmembrane and the cytoplasmic domains of MPL (RWQFP) and has been shown to prevent the dimerization of MPL in the absence of cytokine (8). Its substitution for leucine, lysine, arginine, or alanine leads to constitutive activation of the receptor (58). *MPL* W515L retroviral mouse model after bone marrow transplantation is characterized by a strong thrombocytosis followed by a myelofibrosis recapitulating what is observed in human setting (46). Moreover, a few acquired *MPL* mutations outside the exon 10, leading to amino acid changes in the extra- and intra-cytoplasmic domains of MPL (S204P/F, E230G, Y591D/N, T119I), have been more recently described in sporadic MPN cases. They have been demonstrated to show a lower GOF activity than MPL W515 mutants (59, 60). They could be associated with other acquired or constitutional molecular events, including *JAK2* mutations, to induce the disease phenotype. Finally, the *MPL* S505N mutation was initially described in familial cases of hereditary thrombocytosis, although it has also been described in sporadic cases of ET and PMF (44, 47, 61). S505 is localized in the transmembrane domain of the receptor and the mutation induces a constitutive dimerization of MPL in an active conformation (44).

Other very interesting *MPL* mutations affecting the extracellular domain of MPL (*MPL* P106L and *MPL* K39N) were discovered in hereditary thrombocytosis but are associated with high TPO levels in contrast to other *MPL* mutations.

*MPL* K39N (MPL Baltimore) was described as a polymorphism restricted to 7% of African Americans. Heterozygous cases present a mild thrombocytosis around 500 × 10^9^/L platelets, whereas homozygous cases showed around 800 × 10^9^/L platelets. It was shown that MPL K39N is expressed at low levels at the cell surface and in an incomplete maturation form suggesting a LOF mutation through an abnormal membrane expression of the receptor (48).

*MPL* P106L mutation was initially found in an Arab family, which segregates with strong thrombocytosis in homozygous cases suggesting autosomal recessive inheritance associated with high TPO levels (49). Sometimes, mild thrombocytosis was observed in heterozygous cases. Later on, it was reported that MPL P106L presents a defect in trafficking that prevents its translocation to the cell surface, but is still capable to bind TPO and to signal in the cytosol (62). This seems unlikely since the extracellular domain of MPL is localized in the lumen of endoplasmic reticulum. Our group has also identified such mutation in one Saudian and one Kuweitian family harboring strong thrombocytosis cases with high TPO levels with autosomal recessive inheritance. We confirmed that MPL P106L presents a defect of trafficking, but we demonstrated that low levels of this mutant are addressed at the membrane of MK progenitors. Moreover, there was no spontaneous growth of MKs from patients while MKs, but not platelets, responded to TPO. This study also showed that MPL P106L was better localized at the cell surface on immature than on mature MKs, explaining a proliferative response to TPO in MK progenitors and a defect in TPO clearance in platelets. Moreover, the retroviral *MPL* P106L mouse model performed in *mpl*^−/−^ mice induced a thrombocytosis phenotype with a high TPO level (50). Overall, *MPL* P106L seems to be compatible with an LOF mutation (traffic defect), which induces a paradoxical thrombocytosis through proliferation of MK progenitors and a defect of TPO clearance by the platelet mass.

#### *JAK2* Mutations

The recurrent *JAK2* V617F mutation occurring at exon 14 of *JAK2* was identified in MPNs (34–37) and refractory sideroblastic anemia with thrombocytosis (RARS-T) (63). In MPNs, *JAK2* V617F is present in 50–60% of ET and PMF and 95% of polycythemia vera (PV). The replacement of a guanine to thymidine results in the substitution of a valine by a phenylalanine at position 617, in the pseudokinase JH2 domain of JAK2. The *JAK2* V617F mutation leads to stiffening of the helix alpha C of this JH2 domain, favoring the transphosphorylation of JH1 and thus the constitutive activation of JAK2 (64). The *JAK2* V617F mutation requires the presence of homodimeric type 1 receptors such as MPL, EPOR and G-CSFR to induce a constitutive signaling and a spontaneous proliferation (65). The mutation can be heterozygous or homozygous after a mitotic recombination, with heterozygous clones mainly found in ET while homozygous clones predominate in secondary MF showing a gene dosage effect contribution to the phenotype of the disease (66). In mice, all the models including retroviral, transgenic, and knock-in recapitulate an MPN-like disease (33, 67–70). Even if the most frequent phenotype is a PV progressing to MF, some differences have been observed depending on the species (human or mouse) and on the expression levels of *JAK2* V617F. Particularly, low levels of human *JAK2* V617F in hematopoiesis in inducible transgenic mice with a *jak2* minimal promoter led to an ET, whereas higher expression resulted in PV (71). Moreover, inducible transgenic expression of human *JAK2* V617F only in MK lineage (PF4-Cre) presents a specific ET-like phenotype (72). In knock-in mouse models, germline human *JAK2* V617F led to an ET when heterozygous and to a PV when homozygous (69). The conditional heterozygous mouse *jak2* V617F in hematopoiesis showed an ET/PV phenotype and a severe PV progressing to MF at homozygous state (67, 73). Therefore, as in human setting, the level of *JAK2* V617F determines the disease phenotype.

Other *JAK2* mutations have been highlighted in hereditary thrombocytosis with sometimes the same residue affected than in sporadic MPN cases but with a different amino acid changes, such as *JAK2* V617I, or on different residue located in both the pseudokinase (*JAK2* H608N, *JAK2* R564Q, *JAK2* S755R) and the kinase domain of the protein (*JAK2* R938Q and *JAK2* R867Q) (38–41). All these mutants show a low constitutive kinase activity, seem to trigger spontaneous signaling only in the presence of MPL, and activate more STAT1 than the other STATs in contrast to *JAK2* V617F (41).

#### *THPO* Mutations

Germline GOF mutations in the *THPO* gene have been described in hereditary thrombocytosis. They are located in the 5′-untranslated region or affect splice donor sites of the gene and lead to an increased mRNA translation and synthesis of TPO (54, 55, 74). As an illustration, Figure 2B presents one family with *THPO* mutation predicted to affect the splice donor site of exon 2 (c.13+5G>C) in a hereditary thrombocytosis (Figures 2B,C).

### Mutations on Important Regulators of the MPL/JAK2 Axis

Other molecules are important for the MPL/JAK2 signaling axis and the regulation of megakaryopoiesis.

#### *CALR* Mutations

CALR is a chaperone protein resident of the endoplasmic reticulum. It not only helps neosynthetized proteins and glycoproteins to fold before being addressed to the cell surface but also plays an essential role in calcium homeostasis (75). *CALR* mutations have been identified in ET and PMF in around 25–30% of the cases. These mutations are also, but rarely, found in RARS-T (76, 77). More than 50 different mutations have been described. All of them correspond to frameshift mutations in the exon 9 of the gene and lead to a new and quite similar positively charged C-terminal sequence and the loss of the retrieval KDEL motif into endoplasmic reticulum. The most frequent mutations accounting for more than 80–90% of cases are a 52 bp deletion (type 1 mutation or *CALRdel52*) and an insertion of 5 bp (type 2 mutation or *CALRins5*). These mutations do not have the same prevalence between ET and PMF with the *CALRdel52* more frequently associated with PMF than with ET and conversely for *CALRins5* (78–80). Recently, our laboratory and other groups showed that CALR mutants induce the JAK2/STAT pathway by specifically activating MPL and at a lower extent G-CSFR for the *CALR*del52 mutation only (81). The mechanism is incompletely understood. However, we and others have shown that CALR mutants interact with the glycosylated asparagine in the extracellular part of MPL *via* its lectin domain present in its N-globular domain, but MPL activation requires the new C-terminus and its positive charges (81–84). The retroviral mouse models recapitulate a thrombocytosis progressing into MF more frequently for *CALRdel52* mice than for *CALRins5* mice (84) and the transgenic *CALRdel52* mice recapitulate an ET (85).

#### *LNK* Mutations

Loss-of-function mutations of negative regulators of the MPL/JAK2 signaling axis have also been found to control megakaryopoiesis. SH2B3 (also named LNK) is an adaptor protein, inhibiting the JAK/STAT pathway. It binds to the phosphorylated tyrosine 813 of JAK2 *via* its SH2 domain and controls MPL-mediated signaling (24). *SH2B3* mutations were described in rare cases of ET and PMF patients (86). Most of them occur in a hot spot encompassing residues E208 to G234 of the Pleckstrin (PH) domain, but other mutations are located outside this domain (87, 88). Since this PH domain could be involved in the membrane localization of the protein through the binding to phosphatidylinositol-3-phosphate, the hypothesis is that the mutations detected lead to an abnormal localization of the protein in the cytoplasm (89). However, since the N-terminal dimerization domain remains intact in the LNK mutants, they could bind and interact with the normal (wild-type) protein in the cytoplasm, thus leading to a dominant-negative effect (86). However, the mechanism is still debated since the study of different LNK mutants in the PH domain in cell lines showed a moderate LOF with no dominant-negative effect and an unaffected binding to JAK2 (90). *lnk*^−/−^ mice recapitulate a thrombocytosis associated with a splenomegaly and a MF (91). In MPNs, it is presently unclear if mutations in *LNK* are sufficient to trigger the disease or if another driver mutation such as *JAK2* V617F is absolutely required. Germline mutations in *LNK* have been associated with some primary erythrocytosis (92).

#### *CBL* Mutations

CBL is an E3 ligase, which induces the ubiquitination of MPL and JAK2 and leads to their degradation by the proteasome and the lysosome pathways (22). Mutations in *c-CBL* are nucleotide substitutions or small insertions/deletions in the exons 8 and 9 of the gene and lead to modification of the linker or the RING finger domain (93). They are found in rare cases of ET and PMF. These mutations frequently result in the loss of ubiquitin E3 ligase activity, thus inhibiting lysosome or ubiquitin/proteasome-mediated degradation of MPL/JAK2.

Thus, all the molecules of the TPO/MPL/JAK2 signaling axis are likely to be involved in the MK hyperplasia associated with hereditary thrombocytosis and sporadic classical MPNs. They include GOF mutations of *THPO, MPL, JAK2*, and LOF mutations of *LNK* and *CBL*. These mutations lead to constitutive activation of the downstream signaling pathways, including STAT1/3/5, PI3K/AKT, and MAPK, that control the proliferation/survival and differentiation. Surprisingly, *MPL* LOF can be found in hereditary thrombocytosis by inducing a paradoxical effect on MK progenitors and platelets. Finally, unexpected mutations in *CALR* gene, a chaperone of the endoplasmic reticulum that activates MPL, have also been implicated in sporadic ETs.

## Mutations Involved in Defective Megakaryopoiesis

TPO/MPL axis alterations can also lead to defects in megakaryopoiesis like in some autosomal recessive aplastic anemia and in congenital amegakaryocytic thrombocytopenia (CAMT) (Table 1).

CAMT is a rare autosomal recessive bone marrow failure presenting an isolated thrombocytopenia at birth (94). These cases are associated with very high TPO levels. *MPL* mutations in CAMT are found at homozygous state or compound heterozygous mutations generally located throughout the *MPL* gene, but with a higher recurrence in the exons 2 and 3 encoding the first cytokine receptor motif (52). They can be missense, splicing, frameshift, and nonsense mutations (52, 95). They are LOF mutations, and the frameshift or nonsense mutations are generally more aggressive than missense or splicing mutations leading to early progression to bone marrow failure (52). This is probably due to a complete absence of protein due to truncated proteins or proteins without intracellular signaling. Missense mutations are classified into three groups (Figure 1): (i) those that cannot be addressed to the cell surface and that are blocked in the endoplasmic reticulum, such as *MPL* R102P, *MPL* R257C/L, and *MPL* W154R seem to behave in the same way (51, 95, 96); (ii) those without TPO binding activity due to a loss of stabilization of hydrogen bond, but the MPL mutants, are still normally addressed to the cell surface in a glycosylated mature form, such as *MPL* F104S (19, 52, 96); (iii) those that lead to an unstable protein that is highly degraded, such as *MPL* P635L (51, 97). In *MPL* F104S-like mutation, addition of eltrombopag, a non-peptide TPO mimetic that binds to the transmembrane domain of MPL at histine 499, may overcome the thrombocytopenia (98, 99). In contrast, romiplostim that is a TPO peptide mimetic that binds to MPL in a competitive manner could not be used in this case. In *MPL* P635L-like mutations, some proteasome inhibitors may show benefit by stabilizing the protein. However, in *MPL* R102P-like mutations, the only curative treatment remains the bone marrow transplantation.

*THPO* LOF mutations (Figure 2A) have also been found in families with autosomal recessive bone marrow failure. In one study, a homozygous missense *THPO* R38C mutation was found in two affected siblings localized in the receptor-binding domain that destabilized the interaction between TPO and MPL (11). A recent work has identified three other families carrying two novel mutations *THPO* R99W, *THPO* R157* (57). One case was efficiently treated with TPO mimetics such as romiplostim as a substitution therapy. Alternatively, eltrombopag could also be used (98, 100). Finally, another heterozygous *THPO* R31* was observed but this time in two families of inherited thrombocytopenia. These cases presented with various penetrance of the disease, low TPO levels, and mild thrombocytopenia (100 × 10^9^/L) (56).

*SRC* GOF mutation (*SRC* E527K) has been identified in families with thrombocytopenia progressing into MF and displaying elevated TPO levels. Src family kinases such as BTK and SYK have been shown to interact with MPL at the residue Y591 and participate in its negative regulation since depletion of SRC induces an increase in MPL phosphorylation (101). *SRC* E527K mutation increases the kinase activity of SRC and may negatively regulate MPL activation, which could partially explain the defective platelet production.

## Conclusion

Overall, thrombocytosis and thrombocytopenia have been shown to be due to mutations in molecules of the TPO/MPL/JAK2 signaling axis. In both disease cases, the same molecule can be affected leading to two opposite phenotypes by different mechanisms. Thrombocytosis is either due to GOF alterations that activate MPL signaling (*MPL, JAK2, CALR*) or due to LOF mutations of negative regulators of this pathway (*CBL, LNK*) (Figure 1). In certain cases, GOF mutations in *THPO* lead to increased TPO synthesis and thrombocytosis (Figure 2). Alternatively, thrombocytosis can paradoxically result from mutations in *MPL* that lead to defect in MPL trafficking and result in increased TPO levels by alteration of its clearance. Interestingly, these LOF mutations can thus functionally behave as GOF for platelet production *in vivo*.

## Author Contributions

The authors IP, CBC, MM, SM, CM and WV wrote different parts of the manuscript.

## Conflict of Interest Statement

The authors declare that the research was conducted in the absence of any commercial or financial relationships that could be construed as a potential conflict of interest.
